# Anticancer Applications and Pharmacological Properties of Piperidine and Piperine: A Comprehensive Review on Molecular Mechanisms and Therapeutic Perspectives

**DOI:** 10.3389/fphar.2021.772418

**Published:** 2022-01-07

**Authors:** Sicon Mitra, Uttpal Anand, Niraj Kumar Jha, Mahipal S. Shekhawat, Suchismita Chatterjee Saha, Potshangbam Nongdam, Kannan R. R. Rengasamy, Jarosław Proćków, Abhijit Dey

**Affiliations:** ^1^ Department of Biotechnology, School of Engineering and Technology, Sharda University, Greater Noida, India; ^2^ Department of Life Sciences, Ben-Gurion University of the Negev, Beer-Sheva, Israel; ^3^ Department of Plant Biology and Biotechnology, Kanchi Mamunivar Government Institute for Postgraduate Studies and Research, Lawspet, India; ^4^ Department of Zoology, Nabadwip Vidyasagar College (Affiliated to the University of Kalyani), Nabadwip, India; ^5^ Department of Biotechnology, Manipur University, Canchipur, India; ^6^ Green Biotechnologies Research Centre of Excellence, University of Limpopo, Sovenga, South Africa; ^7^ Department of Plant Biology, Institute of Environmental Biology, Wrocław University of Environmental and Life Sciences, Wrocław, Poland; ^8^ Ethnopharmacology and Natural Product Research Laboratory, Department of Life Sciences, Presidency University, Kolkata, India

**Keywords:** piperine, piperidine, piper, anti-breast cancer, anti-prostate cancer, anti-ovarian effect, mechanism of action, anti-gastric cancer

## Abstract

Piperine and piperidine are the two major alkaloids extracted from black pepper (*Piper nigrum*); piperidine is a heterocyclic moiety that has the molecular formula (CH_2_)_5_NH. Over the years, many therapeutic properties including anticancer potential of these two compounds have been observed. Piperine has therapeutic potential against cancers such as breast cancer, ovarian cancer, gastric cancer, gliomal cancer, lung cancer, oral squamous, chronic pancreatitis, prostate cancer, rectal cancer, cervical cancer, and leukemia. Whereas, piperidine acts as a potential clinical agent against cancers, such as breast cancer, prostate cancer, colon cancer, lung cancer, and ovarian cancer, when treated alone or in combination with some novel drugs. Several crucial signalling pathways essential for the establishment of cancers such as STAT-3, NF-κB, PI3k/Aκt, JNK/p38-MAPK, TGF-ß/SMAD, Smac/DIABLO, p-IκB etc., are regulated by these two phytochemicals. Both of these phytochemicals lead to inhibition of cell migration and help in cell cycle arrest to inhibit survivability of cancer cells. The current review highlights the pharmaceutical relevance of both piperine and piperidine against different types of cancers.

## Introduction

**FIGURE 1:**
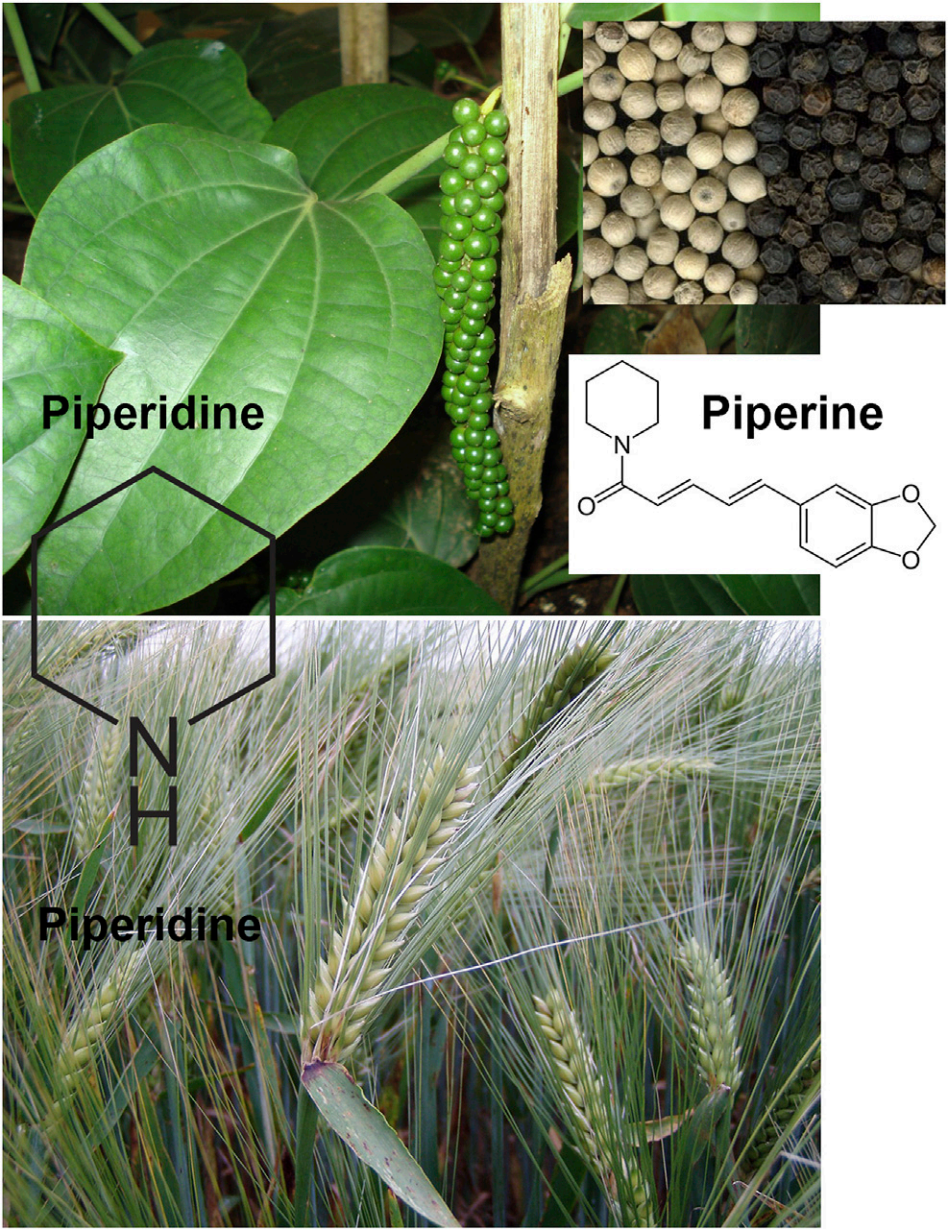
Piperine and piperidine are the two major alkaloids extracted from black pepper (*Piper nigrum* L.). Piperidine can be also found in barley (*Hordeum vulgare* L.). All photographs were reproduced on CC BY-SA 3.0 licence.

Piperidine is a heterocyclic moiety with the molecular formula (CH_2_)_5_NH. It consists of 6 membered rings that further comprises five methylene groups (-CH_2_-) and one amine group (-NH-). This compound is responsible for the synthesis of pharmaceuticals with substantial interest. This compound can be found in barley (*Hordeum vulgare* L., Poaceae) and black pepper (*Piper nigrum* L., Piperaceae, Figure 1).

Piperine, on the other hand, is a piperidine that is substituted by a (1E,3E)-1-(1,3-benzodioxol-5-yl)-5-oxopenta-1,3-dien-5-yl group on the nitrogen atom. It is an alkaloid that has been isolated from the black pepper plant (*Piper nigrum*, Figure 1) and acts as a plant metabolite, an NF-kappaB inhibitor, a human blood serum metabolite and a food component. It is a member of a piperidine alkaloid, benzodioxoles, N-acylpiperidine, and a tertiary carboxamide, which is derived from (E,E)-piperic acid. Piperine and piperidine can perform several other anticancer biological processes, such as ROS release, activation of mitochondrial cytochrome C, release of Bax-protein from mitochondria, and downregulating Bcl-2 protein, resulting in high Bax:Bcl-2 ratio. These processes further result in the activation of caspase-3/9/8 induced cell apoptosis in cancer cells.

To date, black pepper extracted products piperine and piperidine have shown great therapeutic and clinical agents as anticancer potential alone or in combination with other phytochemicals or conventional anticancer drugs; many *in vitro* and *in vivo* studies have shown that piperine and piperidine exhibit several anticancer properties when used against triple negative breast cancer cells (Greenshields et al., 2015; Arun et al., 2018). These two phytochemicals have also shown their anticancer effect against ovarian cancer, prostate cancer, lung cancer and many others which are further discussed in this review paper. Earlier studies have also shown that piperine and piperidine can activate or inhibit several signalling pathways crucial for cancer regulation.

This review summarizes and further discusses the anticancer potential and pharmaceutical properties of piperine and piperidine. The molecular mechanism of action of piperine and piperidine as anticancer molecules, the therapeutic potential of piperine and piperidine when used alone or in combination with other phytochemical molecules against different types of cancer is also discussed. We aim to provide useful information, insights, along with the basis for clinical and therapeutic studies to develop piperine and piperidine as an anticancer drug.

## Methodology of Data Retrieval

This is a review paper in which the already published literature works of different authors are retrieved for review. The source from which the data is collected is mainly journal articles. The main focus is given on the synthesis of relevant literature so that the research aims and objectives can be evaluated. Therefore, the use of this method will provide the reader with a detailed and state-of the-the-art understanding of therapeutic perspectives of piperidine and piperine, along with the anticancer properties of such. To do so, relevant literature works are collected from databases, keeping in mind that obtained and selected resources are available over the Internet and gives better understanding of the topic. It is also emphasized that the collected studies are not older than 1992. A total of 108 publications are retrieved and cited accordingly. With all these, the summarized result of this paper includes 11 different types of cancers for piperine and 5 for piperidine.

## Used Databases and Search Words

One of the most popular academic search engines run by Google, Google Scholar, was used, which indexes a wide range of scholarly literature. The availability of numerous free resources has helped to discover this review of the scientific literature. Additionally, databases such as PubMed, Elsevier, Wiley, and ScienceDirect were used for the retrieval of published studies.

Relevant literature was retrieved using search words and strings, mainly “Piperine,” “Piperidine,” “Piperidine mechanism,” “Piperidine mechanism,” “Piperidine mechanism,” “Piperidine effect against breast cancer,” “apoptosis,” “Ovarian cancer,” “Gastric cancer,” “gliomal cancer,” “lung cancer,” “oral squamous carcinoma,” “chronic pancreatitis,” “prostate cancer,” “leukemia,” and “colon cancer.”

## Anticancer Properties of Piperine and Piperidine: Molecular Mechanisms

Both piperine and piperidine have been reported over the past years to have several efficient pharmacological abilities, including anticancer ability as well. These compounds can activate several molecular pathways, which further leads to apoptosis of cancer cells. Recent observation on the anticancer activity of piperine and piperidine has described its mechanism of activating signaling pathways like NF-κB, PI3k/Aκt etc. which are involved in cancer progression including caspase-dependent pathway to induce apoptosis (Rather and Bhagal, 2018; Zadorozhna et al., 2019). A brief description of the mechanism of piperine and piperidine against different types of cancer is given below.

### Piperine as a Clinical Agent and its Mechanism of Action Against Different Types of Cancer

#### The Anti-breast Cancer Effect of Piperine and its Mechanism of Action

Piperine was found to inhibit cell proliferation of triple negative breast cancer deficient in p53 (TNBC), estrogen receptor positive breast cancer cell, p53 deficient and estrogen receptor positive that express p53 (Huovinen et al., 2011). Functional p53 has been reported to be not required for piperine-mediated growth inhibition. As in most breast cancer cells p53 is very often mutated and does not function properly, in many reports piperine is capable of inhibiting the growth of p53-deficient breast cancer cell lines (Cancer Genome Atlas Network, 2012). Cell proliferation is carried out by completion of cell cycle and expression of many proteins associated with different cell cycle phases are required, such as cyclin D3, E2F-1 and CDK4 for G1, cyclin B, Cdc25C and CDK1 for G2. D-type cyclins are required to bind to CDK4/6 to carry out G1 activity (Malumbres and Barbacid, 2009). E2F-1 is necessary for the G1/S phase transition (Bracken et al., 2004). For the G2/M transition and the G1/S transition Cdc25C, a phosphatase is involved (Malumbres and Barbacid, 2009). p21^Waf1/Cip1^ inhibits CDK activity and when TNBC cells were treated with piperine p21^Waf1/Cip1^, expression was increased (Greenshields et al., 2015). The phosphatidylinositor-3-kinase/Akt signaling pathway is an important pathway for the survival of breast cancer cells (Clark et al., 2002).

When breast cancer cell lines were treated with piperine, the phosphorylation of the Ser473 residue on Akt was decreased, leading to inhibition of the Akt signaling pathway. Furthermore, inhibition of the Akt signaling pathway causes apoptosis of breast cancer cells. Thus, piperine acts as a phosphatidylinositor-3-kinase/Akt and induces apoptosis of breast cancer cells (Greenshields et al., 2015). Recently, the phosphotidylinositor-3-kinase/Akt signaling pathway has been shown to be a clinical agent for the treatment of breast cancer (Zhang et al., 2013).

**FIGURE 2:**
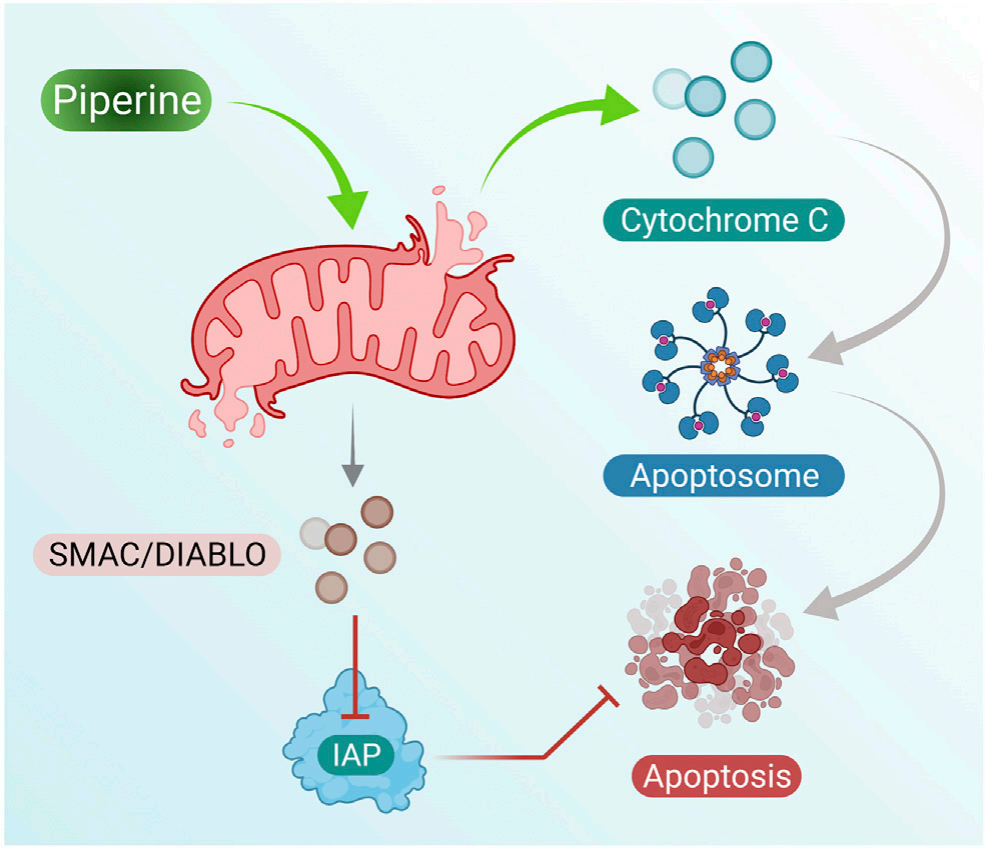
Piperine treatment mediates cytochrome c secretion (activates apoptosome formation) and mitochondrial SMAC/DIABLO, which suppresses IAP activity (inhibits cell apoptosis); these induce cell apoptosis in breast cancer cells.

Piperine treatment of breast cancer cells showed reduced mitochondrial membrane integrity along with the release of cytochrome c and Smac/DIABLO from the mitochondrial cytosol. Furthermore, cytochrome c results in the formation of apoptosomes that result in the activation of the initiator caspase-9 (Twiddy et al., 2004). Inhibitor apoptosis proteins (IAPs) are responsible for inhibition of caspase activity; however, Smac/DIABLO induced by addition of piperine in breast cancer cells inhibits IAPs by binding to them and helps in activation of apoptosis of breast cancer cells (Figure 2 presents the mechanism of piperine releasing mitochondrial cytochrome-c, which further activates apoptosome as well as inhibition of IAP by activation of Smac/DIABLO (Chai et al., 2000).

**FIGURE 3:**
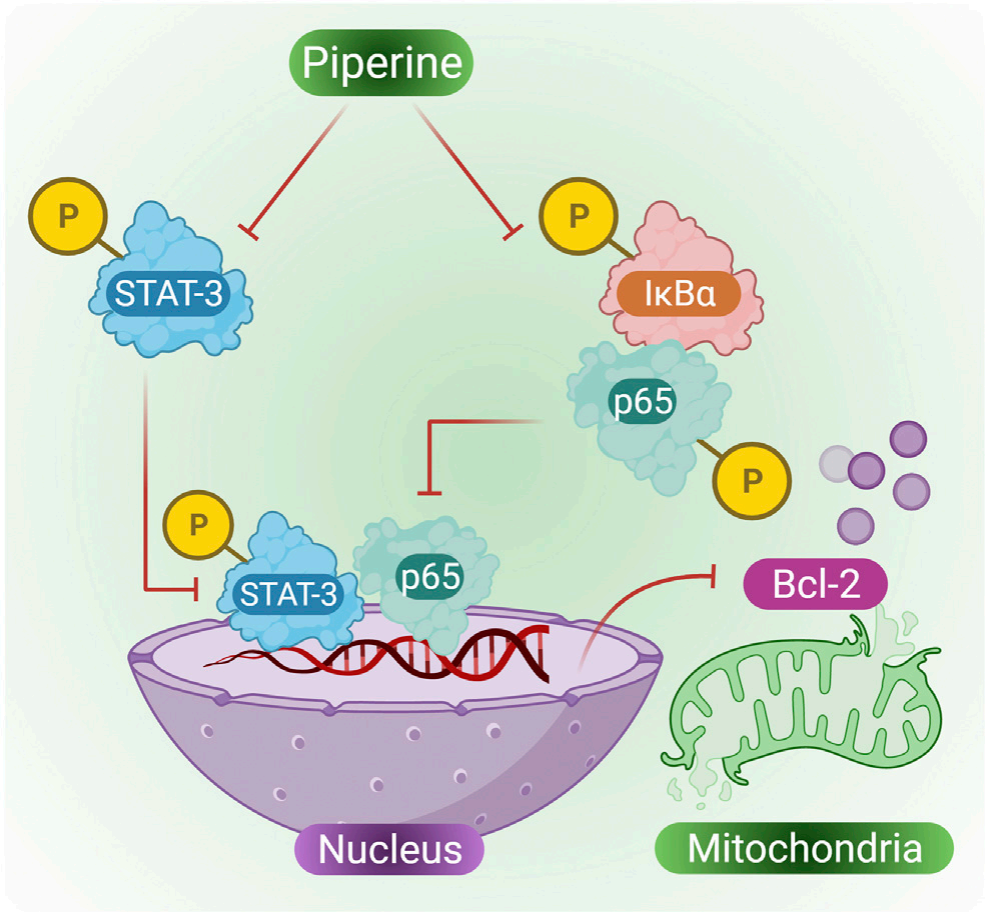
Piperine induces the suppression of STAT-3, IκBα and p65 through phosphorylation, which further inhibits BCL-2 resulting in activation of cell apoptosis in cervical cancer cells.

Expression of the MMP-2 and MMP-9 gene is necessary for induction of metastasis, as a high level of MMP-2 and MMP-9 proteins have been observed in breast tumor tissue (Köhrmann et al., 2009). They are also required for the migration of TNBC. However, when TNBCs were treated with piperine MMP-2 and MMP-9, synthesis was inhibited (Figure 3 presents the mechanism of action of piperine on inhibition of MMP-2 and MMP-9 that results in inhibition of breast cancer cell inhibition) (Greenshields et al., 2015). However in contradiction to this, Sriwiriyajan et al. (2016) reported the potent cytotoxic effects of a piperine-free *Piper nigrum* extract against MCF-7 breast cancer cells and N-nitrosomethylurea-induced mammary tumorigenesis in rats. Further study is needed to assess and compare the anticancer efficacy of both piperine-free plant extract and plant extracts containing piperine with that of pure piperine.

#### Anti-Ovarian Effect of Piperine and its Mechanism of Action

Ovarian cancer is one of the most widespread and deadly gynecological diseases among women around the world (Tai et al., 2012; Chen et al., 2013). Si et al. (2018) studied the effect of piperine on human ovarian cancer A2780 cells, their study confirmed that piperine acts as an antiproliferative agent against human ovarian cancer A2780 cells in a dose and time dependent manner, but piperine did not show any such effect on normal human surface epithelial (OSE) cells (Si et al., 2018). Apoptosis plays a vital morphological process (Danial and Korsmeyer, 2004; Sreedhar and Csermely, 2004). Several apoptotic genes are expressed in uncontrolled proliferative cells that induce apoptosis or programme cell death (Elmore, 2007). Therefore, induction of apoptosis in cancer cells has become an important target for therapeutic drugs over the past years. Piperine treatment in a dose-dependent manner can induce apoptosis in A2780 cells in early and late stages, and this was confirmed by flow cytometric data (Si et al., 2018). Mitochondrial membrane potential is one of the main factors for inducing apoptosis in proliferative cells, using fluorescent dye Rh123 it was found that piperine treatment in A2780 cells can result in loss of Δ*ψ*m leading to cell apoptosis (Guo et al., 2013; Shao et al., 2014).

Furthermore, damage to mitochondrial membrane potential leads to the release of cytochrome c from mitochondria to the cytoplasm, further leading to the activation of two caspase systems called caspase 3 and caspase 9; caspase-3 further triggers proteolytic cleavage of one of its substrates called PARP which induces the activation of apoptosis in cancer cells in a mitochondrial-induced pathway (Li and Dewson, 2015; Zhao et al., 2015; Shen et al., 2016). Caspase-9 activates apoptosis in cancer cells by disrupting the mitochondrial membrane potential (Troy and Shelanski, 2003). Si et al. further supported this, and using western blot analysis, his studies showed piperine treatment resulted in an increased concentration of cytochrome c accumulating in the cytosol of A2780 cells. Furthermore, treatment of A2780 cells with piperine for 48 h resulted in increased activity of caspase-3 and caspase-9, but the level of caspase-8 remained unchanged under similar conditions, which confirmed activation of apoptosis in A2780 cells by piperine through intrinsic apoptotic pathways (Si et al., 2018). Upregulation of caspase-8 leads to another apoptotic pathway called the death receptor-mediated apoptotic or extrinsic pathway which activates several necrotic factors in cancer cells (Wang et al., 2001; Ola et al., 2011). However, an unchanged concentration of caspase-8 in A2780 cells after piperine treatment confirmed that piperine induces the intrinsic apoptosis pathway rather than the death receptor-dependent pathway (extrinsic) in A2780 cells (Si et al., 2018).

Two other apoptotic proteins JNK and p38 MAPK responsible for inducing apoptosis in piperine-treated ovarian cancer A2780 cells were found using western blot analysis (Si et al., 2018). These two proteins carry out several physiological processes such as development, differentiation, and proliferation in ovarian cancer cells (Chen et al., 2016; Liu and Zhou, 2017). Piperine treatment resulted in increased phosphorylation of JNK and p38 MAPK in ovarian cancer cells, which induced apoptosis in A2780 cells suggesting that the intrinsic apoptotic pathway mediated by JNK/p38 MAPK can be induced in ovarian cancer cells by piperine treatment (Si et al., 2018). Piperine inhibited migration in the OVCAR-3 cell line in a concentration-dependent manner along with this it also has antiproliferative effects by apoptotic cell death, caused cell cycle arrest at the G2/M phase, and inhibits the PI3K/Akt/GSK3_ signal transduction pathway (Qiu et al., 2019). It can be concluded that piperine can be used as a clinical agent against ovarian cancer cells.

#### Effect of Piperine on Anti-Gastric Cancer and its Mechanism of Action

Piperine, a major compound extracted from black pepper, has shown its anti-gastric cancer activity in several studies. Ling et al. have confirmed that IL-6 can induce gastric cancer cell invasion by activating the c-Src/RhoA/ROCK signaling pathway (Lin et al., 2007). Piperine was found to inhibit IL-6 expression, further suppressing cell invasion of gastric cancer cells (TMK-1) (Xia et al., 2015). Earlier reports from Łukaszewicz-Zając et al. have shown that high levels of IL-6 are responsible for inducing gastric cancer cells (Łukaszewicz-Zając et al., 2010). Furthermore, activation of the signal transducer and transcription-3 activator (STAT3) resulted in an increased level of IL-6 in gastric tumor cells (Wang et al., 2009). Catherine et al. reported that IL-6 is expressed by IL-1ß through the phosphatidylinositol 3-kinase-dependent AKT/IκB kinase α pathway (Cahill and Rogers, 2008). This was further confirmed by Xia et al. (2015) when IL-1ß increased IL-6 in gastric cancer cells (TMK-1). In TMK-1 cells, IL-1ß can also activate the MAPK pathway, three major MAPK pathways are ERK1/2, p38 MAPK, and SAPK/JNK pathway. However, the p38 signaling pathway is responsible for upregulating the expression of IL-1ß, which further increases the production of IL-6 in TMK-1 cells (Johnson and Lapadat, 2002; Xia et al., 2015). Reports from Xia et al. (2015) confirmed that piperine can inhibit both the STAT-3 pathway and p38 and can be used as an anti-gastric compound against TMK-1 cells.

#### Anti-Gliomal Cancer Effect of Piperine and its Mechanism of Action

One of the most common cancers around the world is glioblastoma multiforme (GBM), it is a malignant brain tumor. One of the most effective medications for clinical GBM therapy is temozolomide (TMZ), which is an alkylating agent and has an imidazole tetrazine ring in its molecular structure (Sansom, 2009). TMZ rapidly penetrates the blood-brain barrier and delays glioma recurrence (Friedman et al., 2000). It can also induce apoptosis by triggering the DNA lesion O6-methylguanine and a transition in p53 function (Roos et al., 2007). Jeong et al. studied the effect of piperine treatment in the U251-MG glioma cell line and reported that piperine can induce TMZ resistant GBM cells that are further sensitive to the drug and compared to treatments with either compound alone, piperine and TMZ together demonstrated inhibition of cell development, indicating that clinically high doses of TMZ alone can possibly be avoided (Jeong et al., 2020). Further study also revealed that low-concentration piperine-TMZ treatment can increase caspase activity (8/-9/-3) that further leads to apoptosis in GBM cells, implying that apoptosis induction in TMZ-resistant GBM cells was regulated by modulating caspases-mediated signaling (Jeong et al., 2020).

CDK-4/6cyclin D and CDK-2cyclin-E complexes have been reported to phosphorylate the Rb protein during the cell division stage, allowing the G1/S transition (Tadesse et al., 2018). After low-concentration piperine-TMZ therapy, RT-PCR of positive cell cycle regulators revealed substantial inhibition of the expression levels of CDK-4/6-cyclin D and CDK2-cyclin-E complexes, implying that G1/S cell cycle arrest occurs; this may be one of the reasons for the increase in the ratio of G1/S (Jeong et al., 2020). Another signaling pathway that is responsible for apoptosis, gliomal development, and other physiological processes is known to be the JNK/p38 MAPK signaling pathway (Tomicic et al., 2015; Si et al., 2018). Furthermore, it has been confirmed by reports from Jeong et al., a low concentration of Piperine-TMZ treatment can induce cell death *via* intrinsic apoptosis in GBM cells by effectively increasing the rate of phosphorylation in the JNK/p38 MAPK signaling pathway (Jeong et al., 2020).

Malignant tumors show an important biological characteristic of tumor migration ability (Demuth and Berens, 2004). Piperine and TMZ alone could not inhibit this ability of tumor migration; however, when piperine and TMZ were treated together in low-concentration tumor migration of gliomal cells were inhibited (Jeong et al., 2020). Therefore, we can conclude that piperine and the TMZ combination exhibit an anticancer effect in gliomal cells by various physiological processes such as inhibiting tumor progression, inducing apoptosis in GBM cells through caspase activity, as well as activation of the JNK/p38 MAPK signaling pathway, so piperine can be used as a clinical agent against gliomal cancer.

#### Anti-Lung Cancer Effect of Piperine and its Mechanism of Action

One of the leading cancers in the world is lung cancer, and its treatment is very limited (Jemal et al., 2006). Lin et al. (2014) performed experiments with the action of piperine on human lung cancer cells (A549) and on human lung fibroblasts (W138) and their findings revealed that piperine can inhibit cell proliferation and can also induce apoptosis in A549. However, piperine did not show toxicity against human lung fibroblasts (W138) (Lin et al., 2014). Piperine was found to express a high level of the p53 gene in a concentration manner. We know that p53 is a tumor suppressor protein that plays a crucial role in inducing cell cycle arrest in the G2/M phase and can also induce apoptosis in cancer cells (Bunz et al., 1998; Karpinich et al., 2002). Thus, a high level of p53 expressed in the presence of piperine could induce cell cycle arrest in the G2/M phase in A549 cells (Lin et al., 2014). P53-dependent apoptosis is regulated by another protein called Bcl-2 (Tian et al., 2008).

Studies have revealed that piperine-treated A549 cells can show an increased level of Bax proteins and a decreased level of Bcl-2 proteins leading to a high Bax/Bcl-2 ratio, which further helps in inducing apoptosis in A549 cells (Lin et al., 2014). Several other important proteins involved in the action of p53-mediated apoptosis are members of the caspase family (Budihardjo et al., 1999). Piperine can induce the activation of caspase-9 and caspase-3 in A549 cells, and thus these two proteins can induce p-53-mediated apoptosis in A549 cells. However, piperine did not activate caspase-8 or caspase-1 in A549 cells (Lin et al., 2014). Thus, these are some signaling pathways through which piperine can inhibit the cell growth, differentiation, and induce apoptosis of lung cancer cell.

#### Effects of Piperine on Oral Squamous Carcinoma

Piperine, a nitrogenous compound, is reported to have anticancer potential against oral squamous carcinoma. Several anticancer actions like nuclear condensation, intracellular ROS generation, depolarization of mitochondrial membrane potential, cell cycle arrest, and caspase activity were observed when the oral squamous carcinoma (KB) cell line was treated with piperine. According to the study of Siddiqui et al. (2017), piperine was reported to act in a dose-dependent manner and has an apoptotic response rather than necrosis and this was further supported by nuclear condensation, caspase-3 activity, and cell cycle checkpoints. Furthermore, when KB cells were treated with piperine for 12 h, an excessive amount of ROS production was observed and this induction of ROS enhancement within the cell helped induce apoptosis in KB cells (Siddiqui et al., 2017). Earlier reports suggest that enhancement of ROS production in glutathione antioxidant system disabled cancer cells, which further leads to apoptosis of such cancer cells (Trachootham et al., 2009). Another factor responsible for apoptosis is the disruption of mitochondrial membrane integrity, which leads to depolarization and induces cell apoptosis (Song et al., 2015; Chaudhary et al., 2016).

Similarly, the depolarization of the mitochondrial membrane potential was observed when KB cells were treated with piperine, which supports the apoptotic action of piperine through mitochondrial membrane depolarization (Siddiqui et al., 2017). The findings of Siddiqui et al. (2017) indicated that piperine inhibits KB (oral squamous carcinoma) growth by inducing cell cycle arrest in the G2/M phase by 15.57 and 37.79% at a concentration of 100 and 200 µM respectively of piperine. This was also accompanied by a reduction in the number of cells in S phase of cell cycle by reducing DNA content, which further led to an increase in the number of apoptotic cells (Siddiqui et al., 2017). Caspase-3 helps in cancer cell apoptosis by cleaving cellular substrates. However, this apoptotic pathway can be either caspase-independent or caspase-dependent signal pathway (Lee et al., 2004). In this context, the study by Siddiqui et al. (2017) has revealed that piperine induces caspase-3 *via* the caspase-dependent pathway, thus promoting apoptosis of cancer cells. Thus, from all the above findings it can be concluded that piperine can be used as a therapeutic agent against oral squamous carcinoma.

#### Effect of Piperine on Chronic Pancreatitis and Mechanism of Action

Chronic pancreatitis (CP) is an injury to the pancreas that leads to inflammation and fibrosis. Piperine has been reported to be a potential therapeutic agent against pancreatitis. The mode of action by which piperine reduced the severe impact of chronic pancreatitis is through inhibition of inflammation and fibrosis by downregulating TGF-ß/SMAD signaling. According to the study by Choi et al. (2019), piperine can inhibit glandular atrophy and inflammatory cell infiltrations, as the well as pancreas of CP-induce mice has witnessed a growth in the number of amylase positive cells, suggesting an improved exocrine action of piperine in acinar cell death and inhibition of inflammation (Choi et al., 2019).

One crucial signaling pathway that plays a role in maintaining the inflammatory response, cell growth and differentiation, and immune system is TGF-ß/SMAD signaling (Prud'Homme, 2007). Pancreatic stellate cells (PSC) after sustaining cell injury can release many pro-fibrogenic factors which can promote fibrotic responses, one such factor being TGF-ß induced pancreatic fibrogenesis (Schneider et al., 2001; Apte et al., 2012). Piperine can also regulate fibrosis by inhibiting the expression of fibronectin-1, collagen I/III and α-SMA upon treatment of TGF-ß, thus piperine can negatively regulate the activation of PSC (Choi et al., 2019).

Furthermore, SMAD proteins are activated by phosphorylation in the TGF-ß signaling pathway which is then translocated into the nucleus where they form complexes with transcription factors and regulate the expression of profibrotic genes (Wynn, 2008; Fabregat et al., 2016). Studies have revealed that PSCs are activated by the TGF-ß pathway in a SMAD2/3 dependent manner (Ohnishi et al., 2004). However, piperine does not inhibit pSMAD1/5 in PSC (Choi et al., 2019). Thus, it can be concluded that piperine plays a crucial anti-pancreatic role in pancreas stellate cells by downregulating several pathways leading to activation of chronic pancreatitis and piperine tends to have an antifibrotic effect so it can be used as a clinical agent against pancreatic disorders.

#### Anti-Prostate Cancer Effect of Piperine and its Mechanism of Action

Recent studies show that the number of men between the age group of 15–65 has been suffering from prostate cancer (Quon et al., 2011). Studies have revealed that piperine can exhibit its antiproliferative activity in androgen-dependent (AD) LNCaP and androgen-independent (AI) PC-3, DU-145 and 22RV1 PCa cells. However, the sensitivity of LNCaP towards piperine was found to be higher, followed by PC-3, 22RV1, and DU-145. Apoptosis in prostate cancer cells is executed by the caspase-3 enzyme (Gnanasekar et al., 2009). Piperine is effective in activating caspase-3 in prostate cancer cells. Another antiapoptotic factor found in prostate is STAT-3; it helps in the induction of cancer in prostate (Huang et al., 2000; Guo et al., 2011; Doucette et al., 2012). Therefore, from studies by Samykutty et al. piperine was found to be responsible for inhibition of STAT-3 phosphorylation activation in DU145, PC-3 and LNCaP. Inhibition of STAT-3 further results in inhibition of prostate cancer cell growth (Samykutty et al., 2013). Piperine significantly inhibited cell migration in the DU145 prostate cancer cell line (Zeng and Yang, 2018).

We know NF-κB signaling pathway is a major signaling pathway in induction of prostate cancer, cell differentiation, and cell growth. Samykutty et al. have reported that piperine can down-regulate both NF-κB in DU-145 and PC-3 (AI). Similarly, piperine can also reduce expression of NF-κB in LNCaP (AD) (Samykutty et al., 2013). Piperine has other effects in inhibition of prostate cancer cells, such as it can result in cleavage of PARP-1 (Yoo et al., 2008). This can reduce the chances of prostate cancer. Piperine also inhibits B16-F10 invasion of B16-F10 *via* inhibition of NF-κB (Pradeep and Kuttan, 2004). Therefore, piperine *via* PARP-1 cleavage, inhibition of NF-κB, downregulation of STAT-3, and activation of caspase-3 can inhibit prostate cancer and cell proliferation.

#### Anti-Rectal Cancer Effect and Mechanism of Piperine

The role of piperine as anti-rectal cancer has been studied in HRT-18 human rectal adenocarcinoma cells and piperine extracted from black pepper was reported to exert an inhibitory effect on the metabolic activity of HRT-18 cells (human rectal adenocarcinoma) (Yaffe et al., 2013). According to a study by Sherr and Roberts (1999), HRT-18 cells undergo a 40% reduction in cell division when treated with piperine, this suggested the inhibitory effect of piperine on cell cycle progression of HRT-18 cells, in the G_0_/G_1_ phase, the number of associated cells was found to increase, while cells associated with the G_2_/M phase were found to be greatly reduced; this suggested inhibitory effects of piperine of different cyclin dependent kinases such as CDK4 and CDK6 as well as on D-type cyclins (Sherr and Roberts, 1999).

Loo (2003), reported that activation of redox-sensitive transcription factors and the production of a large amount of H_2_0_2_ resulted in proliferation of cancer cells (Loo, 2003). Earlier, it has been reported that piperine plays a role in ROS scavenging (Mittal and Gupta, 2000). Furthermore, piperine-induced apoptosis of HRT-18 rectal carcinoma cell apoptosis by piperine was reported through the action of increasing ROS production (Yaffe et al., 2013). However, piperine production of superoxide anion was reported to be minimal in HRT-18 cells and these cells were found to produce hydroxyl radicals; this suggested the role of piperine in superoxide scavenging. Although these piperine treated cells can also produce a large amount of hydroxyl radical production *via* the Fenton reaction in a dose-dependent manner (Mittal and Gupta, 2000). Skrzydlewska and Farbiszewski (1999) confirmed the involvement of ROS in piperine-induced cell apoptosis, HRT-18 cells were treated with antioxidant NAC which helped to eliminate ROS and also promoted intracellular glutathione synthesis which further resulted in reduced apoptosis of piperine-treated rectal carcinoma cells (HRT-18) (Skrzydlewska and Farbiszewski, 1999). Furthermore, apoptosis of piperine-treated HRT-18 cells *via* caspase activation was reported (Reubold and Eschenburg, 2012). Thus, it can be concluded that piperine can inhibit cell cycle progression, increase ROS production, and induce cell apoptosis in rectal cancer cells; this suggests that piperine can be used as therapeutic agent in rectal cancer. A recent study by de Almeida et al. (2020) reported inhibition of the Wnt/catenin pathway in HCF116 colorectal cancer cell lines by piperine, as well as overexpression of β-catenin, β-cateninS33A or dnTC4FP16, and suppression of β-catenin nuclear localization. In colorectal cancer cells, the Wnt/-catenin signalling pathway and cell movement were inhibited. Piperine prevented cancer cell motility and invasion, reversed epithelial-to-mesenchymal transition biomarker expression, and downregulated STAT3 expression in another colorectal cancer investigation (de Almeida et al., 2020; Song et al., 2020).

#### Effect of Piperine on Anti-Cervical Cancer and its Mechanism of Action

**FIGURE 4:**
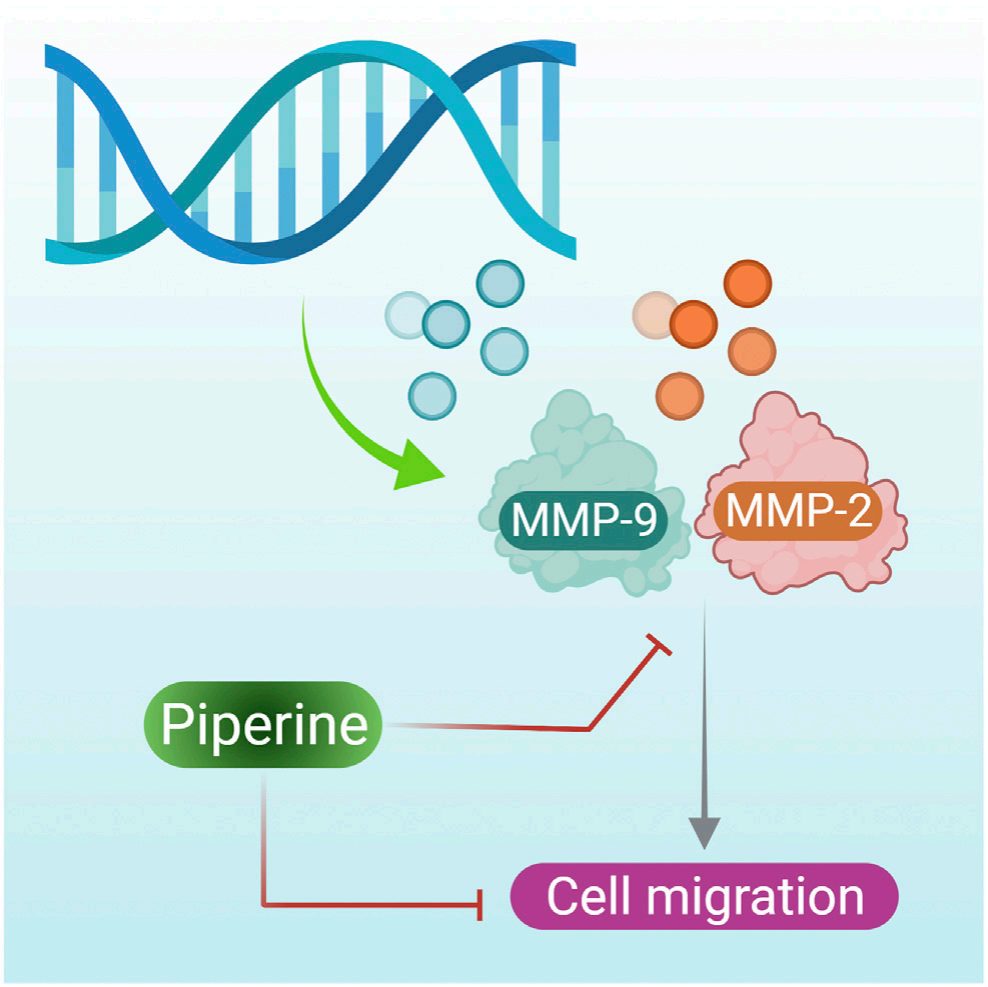
Piperine inhibits the expression of the MMP-2 and MMP-9 gene, further reducing the cell migration of breast cancer cells.

Cervical cancer is one of the most common cancers suffered by women that is approximately 7.5% of all cancers in women (Pfaendler et al., 2015). Previously mitomycin C (MMC) was used as an anticancer antibiotic against cervical cancer; however, over time, cervical cancer cells were found to exhibit multidrug resistant (MDR) and also resistant to MMC. Furthermore, piperine extracted from *Piper longum* L. and *Piper nigrum* was used to study its effect on MMC resistant cervical cancer cells. Co-treatment of MMC and piperine in cervical cancer cells in a dose-dependent manner was found to suppress cell proliferation and promote many cytotoxic effects on MMC cells. According to the study ofHan (2011), indicated that when cervical cells were treated with piperine they showed MMC-induced apoptosis in HeLa cells that were previously resistant to MMC, many other biological effects were also observed, such as decreased expression of p-STAT3, Bcl-2 and NF-κB, however, some biological pathways were also enhanced, such as increased expression of Bax, PARP activity, caspase activity and Bid in HeLa/MMC cells (Han et al., 2017). Reports have confirmed the existence of a cross-talk between NF-κB and STAT3 (He et al., 2010). Both STAT3 and NF-κB plays role in cancer development and progression, STAT3 also regulates the expression of various cancer-causing genes in response to cancer-inducing stimuli (Li et al., 2013). Piperine in combination with MMC is capable of suppressing the expression of STAT3, p65 expression in the nucleus, p-IκB in the cytoplasm, and also plays an important role in suppressing HeLa/MMC cells (Figure 4 presents phosphorylating suppression of STAT3 and p65 in the nucleus and suppression of p-IκB in the cytoplasm by the action of piperine, thus inhibiting mitochondrial Bcl-2) (Li et al., 2014).

Cell apoptosis has been regulated by the expression of the Bcl-2 and Bax proteins, Bcl-2 resists cell apoptosis and induces tumorigenesis, while the expression of Bax results in apoptosis of cancer cells (Davoudi et al., 2014). Thus, the low ratio of Bax to Bcl-2 is a crucial point in induction of cancer cell cycle progression, and the high expression of the ratio leads to potential cell apoptosis as well as results in the release of cytochrome c from mitochondria (Chen et al., 2012). Studies have confirmed that piperine has the potential to reduce Bcl-2 expression and improve Bax expression, thus maintaining a high Ba:Bcl-2 ratio which further increases the amount of cytochrome-c released from mitochondria into the cytosol (Han et al., 2017).

Another important factor essential for the induction of apoptosis in cervical cancer cells is caspase activation (Kaminskyy et al., 2012; Rinwa et al., 2013). According to the study of Han 2011, piperine along with MMC can induce apoptosis by caspase activity in a caspase-3, caspase-8, and caspase-9 dependent manner, resulting in PARP activity that led to apoptosis, cytochrome-c released by piperine activity and MMC also helps to activate caspase-dependent apoptosis (Han et al., 2017). Thus, piperine co-treated with MMC can act as a clinical agent in response to cervical cancer.

#### Mechanism of Action of Piperine Against Leukemia

One of the most widespread and fatal diseases is considered to be leukemia and it has a very high death rate throughout the world (Deschler and Lübbert, 2006). About 3% of all malignancy diseases are contributed by leukemia, and very few patients survive leukemia (Zhang et al., 2010). In search of less toxic treatment for it, several reports over the past years have confirmed positive effects of piperine against leukemia. Li et al., subjected HL60 leukemia cells to piperine and curcumin treatment and observed that both compounds can exhibit several antitumor activities in human leukemic cells (HL60), while these compounds have shown low toxicity in leukemic cells (Li et al., 2020). Two important mechanisms by which piperine induces elimination of malignant cells are apoptosis and autophagy (Lowe and Lin, 2000; Mathew et al., 2007). Western blot analysis by Li et al., confirmed that piperine and curcumin in a dose-dependent manner are capable of elevating the expression level of Bax protein and downregulating the expression of Bcl-2 in human leukemic cells HL60 (Li et al., 2020). As we know, a high Bax:Bcl-2 ratio induces apoptosis in cancer cells, thus piperine can induce apoptosis in HL60 by regulating the level of Bax and Bcl-2 proteins.

Two extrinsic and intrinsic pathways are responsible for inducing apoptosis in cancer cells. The intrinsic pathway starts from the disruption of the mitochondrial membrane potential which releases cytochrome c, which further activates a caspase signaling pathway. Cytochrome c activates caspase 9 which in return activates caspase-3; the increase in caspase-3 concentration results in proteolytic cleavage of PARP which induces cell death, while caspase-8 is responsible for the extrinsic apoptotic pathway (Li and Dewson, 2015; Zhao et al., 2015; Shen et al., 2016). Cotreatment with piperine and curcumin in HL60 cells marked an increase in caspase-3 concentration, thus it can be confirmed that piperine can activate the intrinsic apoptotic pathway in leukemic cancer cells in a dose-dependent manner (Li et al., 2020).

Furthermore, piperine curcumin cotreatment arrests leukemic cells HL60 in the S phase of the cell cycle, which was supported by investigations of Li et al., when his study found an increased number of S phase cells when HL60 were piperine treated; his investigations also reported that piperine along with curcumin can inhibit invasion and migration of HL60 by the wound healing mechanism, suggesting antimetastatic potential of piperine (Li et al., 2020). From all these investigations, it can be concluded that piperine when co-treated with curcumin inhibits the growth and development of leukemic cells through the induction of apoptosis, autophagy, inhibition of cell migration and cell cycle arrest, thus piperine can be used as a potential antileukemic clinical agent. A recent study by Benerjee et al. (2021) confirmed the ability of black pepper and its main constituent piperine to inhibit cellular proliferation of human leukemic cells, K-562 cells. Both exhibited potent cytotoxic effects by upregulating BAX, caspase-3, and caspase-9 while downregulating the Bcl-2 gene.

### Piperidine as a Clinical Agent and its Mechanism of Action Against Different Kinds of Cancer

#### The Anti-Breast Cancer Effect of Piperidine and its Mechanism of Action

According to the study by Arun et al. (2018), the piperidine derivative 1-(2-(4-(Dibenzo[b,f]thiepin-10-yl)phenoxy)ethyl)piperidine (DTPEP) was synthesized from Tamoxifen (TAM). The effect of DTPEP has been observed in both Estrogen Receptor (ER) negative cells (MDA-MB-231) and ER positive cells (MCF-7). DTPEP was found to inhibit cell proliferation in both MDA-MB-231 and MCF-7by restricting the cell cycle in the G0/G1 phase (Arun et al., 2018). We know that the generation of ROS is necessary to induce apoptosis and disruption of mitochondrial membrane integrity, and DTPEP was found to increase ROS synthesis in MDA-MB-231 and MCF-7 cells. DTPEP has also been reported DTPEP results in increased synthesis of cytochrome C and Bax and decreased synthesis of the bcl-2 protein in MDA-MB-231 and MCF-7 cells.

In MCF-7, ER_α_ plays a critical role in the establishment of breast cancer. However, ER_α_ level was reported to be decreased by DTPEP by enhancing ERß levels in ER positive cells. In ER negative MDA-MB-231 DTPEP carried out inhibition of the PI3K/AKT signaling pathway by down-regulation of phosphorylation at tyr485 of PI3K and at ser473 of AKT (Arun et al., 2018). Furthermore, DTPEP was also reported to decrease the protein level of PKCα in both ER positive and ER negative cells (Arun et al., 2018). Thus, DTPEP, which is a piperine derivative, has dual activity in anti-breast cancer cells.

#### Anti-Prostate Cancer Effect of Piperidine and its Mechanism of Action

According to previous data available, prostate cancer is considered to be the second most common cancer in men worldwide (Jemal et al., 2010). The initiation and progression of prostate cancer has been reported to be modulated by androgen receptor (AR) signaling. Thus, targeting AR has been considered a therapeutic option for the treatment of prostate cancer. Several piperidine derivatives were synthesized among them one such derivative is compound 17a, which shows good anti-prostate cancer activity. Fu et al. (2020) studied the effect of compound 17a on PC3 (prostate cancer cell line) and found that it inhibits PC3 proliferation in a concentration-dependent manner. Compound 17a also resulted in inducing apoptosis in PC3 cells by decreasing the expression level of XIAP and BCL-2 and increasing the level of BAX accordingly in a concentration-dependent manner (Samykutty et al., 2013). The epithelial-mesenchymal transition (EMT) is a crucial regulatory step in prostate cancer that is activated during cancer migration (Gnanasekar et al., 2009). A further study also revealed the impact of compound 17a on the epithelial-mesenchymal transition; compound 17a has been stated to hinder PC3 cell migration through the biological membrane and can also upregulate E-cadherin, which is a biomarker for epithelial cells. Meanwhile, N-cadherin and vimentin, which are biomarkers of mesenchymal cells, have been down-regulated by the presence of compound 17a, a piperidine derivative. 17a has also been revealed that can bind to the colchicine binding site and inhibit tubulin polymerization (Samykutty et al., 2013). Thus, it has been revealed that 17a can binds with colchicine binding site and inhibit tubulin polymerisation.

#### Effect of Piperidine on Colon Cancer

Colon cancer is referred to as one of the common malignant tumors of the digestive tract that can be treated with a combination of radiochemotherapy and immunotherapy. This type of cancer has widespread metastasis to the pancreas, lungs, and lymph nodes with poor clinical treatment options. Treatment involving 2-amino-4- (1-piperidine) pyridine was found to prohibited proliferation of HT29 and DLD-1 cells in a dose-based manner (Yaffe et al., 2015). This compound further inhibits progression to the S phase by arresting the DLD-1 and HT29 cell cycle in the G1/G0 phase (Wang et al., 2019). The study by Wang et al. (2019) depicted that DLD-1 and HT29 cells’ ability to migrate and invade was inhibited with treatment with 2-amino-4-(1-piperidine) pyridine (Wang et al., 2019). NDRG2 expression, which is a protein-coding gene, is activated by the transcription factor KLF4 and further helps suppress tumor and exhibits an inhibitory effect on colon cancer cell proliferation (Ma et al., 2017). MACC1, the MET transcriptional regulator, also promotes metastasis and recurrence of colon carcinoma by activating the HGF/c-Met signaling pathway.

However, the specified treatment reduces the expression of FOXA2 mRNA in DLD-1 and HT29 cells, where the protein assists in the proliferation and metastasis of colon cancer cells. The CDH1 gene generates epithelial cadherin or E-cadherin, which helps to inhibit tumor cell invasion. Induction of EMT is the initial mechanism of a complicated process of cell metastasis that promotes tumor progression with cell migration and invasion. EMT and suppression of E-cadherin are a part of E-cadherin expression that enhances the metastatic potential of epithelial tumors. Therefore, the use of amino-4-(1-piperidine) pyridine in the treatment of DLD-1 and HT29 cells down-regulates EMT. Additionally, western blot was evaluated in the vimentin expression at 72 h of treatment with 40, 50 and 100 µM of 2-amino-4-(1-piperidine) pyridine and suppressed in colon cancer cells (Wang et al., 2019).

#### Anti-Lung Cancer Effect of Piperidine and its Mechanism of Action

An antiproliferative compound known as CLEFMA has recently been recognized as an anticancer compound (Lagisetty et al., 2010). CLEFMA 4-[3,5-bis(2-chlorobezylidene)-4-oxo-piperidine-1-yl]-4-oxo-2-butenoic acid is a piperidine derivative that resembles chalcones with a core of diphenyl dihaloketone. CLEFMA has been used to treat lung adenocarcinoma cells (H441 and A549), as well as its impact on normal lung cells (CCL151) has also been observed, and CLEFMA has been reported to induce redox homeostasis in H441 cells, but does not affect normal lung cells (CCL151) (Sahoo et al., 2012). According to reports by Yadav et al. (2013) CLEFMA can induce cell death in lung adenocarcinoma cells *via* many pathways, one such pathway is intrinsic apoptosis that begins with the activity of caspase 9 that further cleaves and activates procaspase-3 and procaspase-9 (Yadav et al., 2013).

Furthermore, the regulation of PARP other cellular targets is carried out by cleavage activity of procaspase-3 and procaspase-9. PARP plays a role in DNA repair, but caspase-mediated cleavage of PARP inactivates it and induces apoptosis (Lagisetty et al., 2010). CLEFMA can induce apoptosis by reducing the concentration of BID and increasing that of BAX, thus maintaining a high ratio of BAX:BID. Few proteases that can cleave and decrease the activity of BID are granzyme B, cathepsins and calpains; further cleaved BID migrates to mitochondria and where it becomes permeable to outer mitochondrial membrane which is dependent on BAX protein (Billen et al., 2008). P53 phosphorylation is another process through which CLEFMA can induce cell death in lung cancer cells (Lagisetty et al., 2010). Kasinski et al. (2008) confirmed that synthetic and natural chalcones can inhibit tumor by inhibiting NF-κB activation (Kasinski et al., 2008). However, CLEFMA did not show such a reduction in NF-κB expression levels in normal lung cancer cells (CCL151) (Lagisetty et al., 2010).

Furthermore, the mechanism of NF-κB signaling pathway in tumor suppression was revealed; during the normal scenario, phosphorylation of IκBα leads to its proteasome-mediated degradation and NF-κB is released to get translocated into the nucleus for its expression. However, in lung cancer cells (H441) CLEFMA inhibited the degradation of phosphorylated IκBα *via* proteasome inhibitors thus NF-κB was inhibited from translocating to the nucleus for expression, further induced cell apoptosis (Traenckner et al., 1994; McDade et al., 1999; Lagisetty et al., 2010). The synthesis of PGE_2_ is catalyzed by COX-2 and is a critical step in the inflammatory pathway. COX-1 is found to be expressed in various cells; however, COX-2 are expressed only in inflammation sites by pro-inflammatory cytokines (Seibert and Masferrer, 1994). These pro-inflammatory cytokines, together with COX-2 expression, were found to be suppressed by CLEFMA, thus inhibiting inflammation (Lagisetty et al., 2010). In addition, another function of NF-κB is to regulate the expression of cyclin D which performs the phase transition from G1 to S in the cell cycle, CLEFMA treatment on lung cancer cells H441 suppress NF-κB, which further leading to reduced expression of cyclin D resulting in the arrest of phase S of H441 cells (Lagisetty et al., 2010). A protein called CD31 is known to be a marker of angiogenesis and tumors; these molecules are expressed in granulocytes and monocytes. CLEFMA treatment suppressed CD31 expression in granulocytes and monocytes, reducing lung cancer cell inflammation. ICAM1 responsible for cell adhesion and migration is also down-regulated by CLEFMA (Lagisetty et al., 2010). Thus, it can be concluded from all this evidence that the piperidine derivative CLEFMA can be used as a clinical agent against lung cancer due to its anticancer effects.

#### Anti-Ovarian Cancer Effect of Piperidine and its Mechanism of Action

Among all gynecological tumors, ovarian cancer is considered to be one of the major tumors that form diseases affecting female reproductive organs (Salehi et al., 2008). However, over the years, proper treatment of ovarian cancer was restricted by the multi-drug resistant capacity of ovarian cancer cells (Markman, 2008). One of the common chemotherapies used in ovarian cancer is cisplatin (DDP), but it often imparts cytotoxic effects at high concentration doses. Therefore, one of the piperidine nitroxide derivative molecules known as tempol (TPL) was studied for its effect on ovarian cancer cells (Wang et al., 2018). Piperidine extracted from black pepper can be used to synthesize TPL, which has an unpaired electron and has three reversible forms: Hydroxylamine, nitroxide, and oxoammonium cation (Krishna et al., 1992). The reduction and oxidizing ability of TPL makes it a suitable agent for use against ovarian cancer (Wilcox and Pearlman, 2008). Treatment with DPP alone cannot show satisfactory effects on ovarian cancer. However, TPL being a redox agent can protect organs from oxidative damage by inhibiting the development of neoplastic cells (Gariboldi et al., 1998). When TPL and DDP were cotreated in ovarian cancer cells (OVCAR3), significant effects were observed; one such effect observed by the MTT assay was inhibition of OVCAR3 cell proliferation. Further flow cytometric results also marked an increase in apoptosis in OVCAR3 cells after co-treatment with TPL and DDP (Wang et al., 2018).

Disruption of mitochondrial membrane potential often leads to inhibition of the electron transport chain system, and this is linked with cell apoptosis induced by ROS generation (Perrone et al., 2008). Furthermore, ROS helps cytochrome c to be released from mitochondria and accumulates in the cytosol, leading to the induction of the mitochondrial apoptotic pathway (Circu and Aw, 2010). The Bax and Bcl-2 ratio is very crucial for the induction of apoptosis in cancer cells, a high ratio of Bax:Bcl-2 regulates the mitochondrial pathway which further leads to activation of caspase-dependent apoptosis as well as the caspase-independent apoptotic pathway (Todorova et al., 2004). Western blot analysis of cotreated OVCAR3 with TPL and DDP has shown upregulation of Bax proteins and a gradual decrease in the concentration of Bcl-2 protein, leading to a high Bax:Bcl-2 ratio (Wang et al., 2018).

**FIGURE 5:**
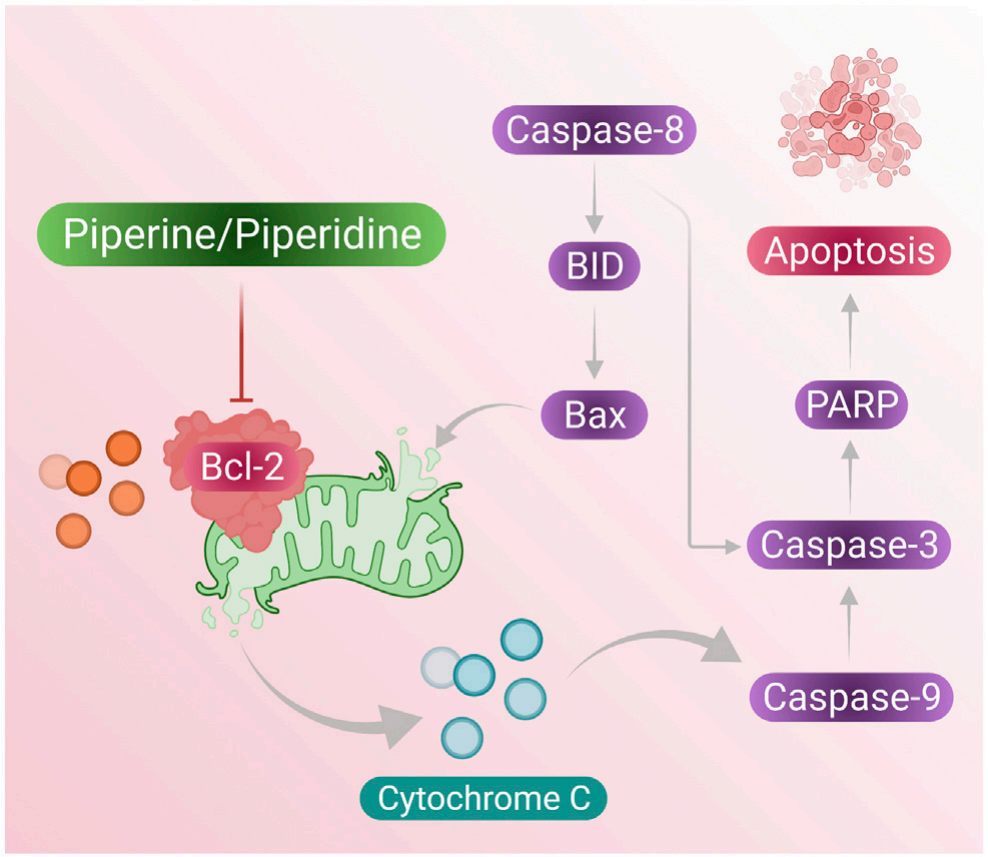
Piperine and piperidine-induced caspase pathway for activating cell apoptosis in cancer cells.

According to reports from Meng Wang et al., reported that DDP-induced TPL can increase ROS generation at the cell level and this was confirmed by the DCFH-DA staining assay, indicating that TPL and DDP together can induce apoptosis in OVCAR by increasing cellular ROS generation (Wang et al., 2018). In conclusion, piperidine derivative nitroxide tempol (TPP) can play some major roles in the induction of apoptosis in ovarian cancer cells when co-treated with DPP and can be a crucial clinical agent in therapeutic treatment. Figure 5 presents overhaul mechanism of the action of piperine and piperidine induced caspase pathway to activate apoptosis in cancer cells. A summary of the treatment effects of piperine and piperidine alone as well as cotreatment with other phytochemicals on existing chemotherapeutics is shown in Table 1.

**TABLE 1:** Treatment and co-treatment effects of piperine and piperidine on the anticancer potential of conventional chemotherapeutics.

Cancer (cells/tumor type)	Phytochemical used	Observations	References
Breast cancer (Triple negative breast cancer cells)	Piperine	Piperine treatment inhibits the growth of p53 deficient cell lines by inhibiting the G1-S transition of the cell cycle and also enhances the expression of p21^Waf1/Cip1^, further inhibiting CDK activity. Piperine resulted in a decrease in the phosphorylation of the Ser473 residue in Akt, leading to apoptosis. Piperine-induced release of mitochondrial Smac/DIABLO, which inhibits IAP (inhibitor of apoptosis) and cytochrome c, which induces apoptosome formation, leading to cell apoptosis. Cancer cell migration is also reduced by piperine-mediated reduced gene expression of MMP-2 and MMP-9	Greenshields et al. (2015)
Ovarian cancer (A2780 cells)	Piperine	Piperine treatment for 48 h resulted in an increase in mitochondrial cytochrome c release which increased caspase-9 and caspase-3 activity (intrinsic pathway), but no such change in caspase-8 concentration has been observed. Caspase-9/3 further initiates cell apoptosis. Phosphorylation of JNK and p38 MAPK by piperine treatment also resulted in an increased rate of apoptosis in A2780 cells	Si et al. (2018)
Gastric cancer (TML-1 cells)	Piperine	Piperine treatment can inhibit IL-6 expression by down-regulating the c-Src/RhoA/ROCK signalling pathway. Furthermore, piperine can also inhibit STAT3 activation. Thus, by inhibiting both STAT3 activation and the expression of p38 piperine can inhibit the gastric properties of TML-1 cell lines	Xia et al. (2015)
Gliomal cancer (U251-MG glioma cell line)	Piperine and Temozolomide (TMZ)	Cotreatment of piperine and TMZ in a low concentration induces several inhibitory effects on the growth of U251-MG cell lines. Piperine and TMZ together can inhibit G_1_/S cell cycle progression by suppressing the expression of cyclin D-CDK-4/6 and cyclin E-CDK2; these two compounds can also initiate apoptosis through the caspase 8/9/3 pathway. Co-treatment also resulted in increased phosphorylation of the JNK and p38 MAPK signaling pathway, which further contributes to cell apoptosis	Jeong et al. (2020)
Lung cancer (A549 cells)	Piperine	Piperine treatment results in the expression of a high level of p53 that leads to the arrest of the phase G2-M in the cell cycle, piperine also reduces the level of Bcl-2 and increases the level of Bax-2 and this high Bax:Bcl-2 ratio helps in the further initiation of caspase 9/3 dependent apoptosis in A549 cells	Lin et al. (2014)
Oral squamous carcinoma (KB cell line)	Piperine	Treatment of KB cells for 12 h marked a high amount of ROS synthesis, which induces cell apoptosis in cancer cells. Piperine at a concentration of 100 and 200 µM leads to cell cycle arrest in the G2/M phase by 15.57 and 37.79%, respectively. Furthermore, piperine treatment also leads to induction of apoptosis by activating the caspase-3 pathway	Siddiqui et al. (2017)
Chronic pancreatitis [PSC (Pancreas stellate cells)]	Piperine	Piperine treatment can down-regulate the TGF-ß/SMAD signaling pathway along with this piperine can also inhibit the expression of fibronectin-1, collagen I/III and α-SMA. Inhibition of all of these pathways leads to inhibition of the cancer potential of PSC.	Choi et al. (2019)
Prostate cancer (LNCaP, PC-3, DU-145 and 22RV1 PCa cells)	Piperine	Piperine treatment on these cell lines can effectively activate the caspase-3-dependent apoptotic pathway. Piperine-mediated inhibition of cell growth was observed by suppressing activation of phosphorylated STAT-3 in DU145, PC-3, and LNCaP cells. Treatment with piperine can also down-regulate the expression of Nf-κB in DU145, PC-3 and LNCaP and can also induce PARP-1 cleavage. All of these processes along with inhibition of B16-F10 invasion results in the anticancer potential of piperine against prostate cancer cells	Samykutty et al. (2013)
Rectal cancer [Human rectal adenocarcinoma cells (HRT-18)]	Piperine	Piperine treatment reduces HRT-18 cell division by 40%. Piperine results in inhibition of G_0_/G_1_ cell cycle progression by down-regulating the synthesis of CDK4/6 and cyclin D. Enhanced ROS and other superoxide anion synthesis is also an outcome of piperine treatment. Thus, ROS synthesis and inhibition of cell cycle progression result in apoptosis of cancer HRT-18 cells	Yaffe et al. (2013)
Cervical cancer (HeLa/MMC cells)	Piperine and Mitomycin-C (MMC)	Co-treatment of piperine and mitomycin C (MMC) induced apoptosis of HeLa cells along with several other biological effects such as decreased expression of p-STAT3, Bcl-2, NF-κB and increased expression of Bax, Bid, PARP activity, and caspase 3/9 and caspsae-8 induced cell apoptosis of HeLa/MMC cells. All of these biological effects of piperine exhibit its anticervical cancer potential when used along with Mitomycin C	Han et al. (2017)
Leukemia (HL60)	Piperine and curcumin	Combined treatment of piperine and curcumin in HL60 cells results in many anticancer biological effects, such as the establishment of a high Bax:Bcl-2 ratio by increasing Bax synthesis and downregulating Bcl-2 expression level, and release of mitochondrial cytochrome-c, which further initiates caspase-9/3 mediated cell apoptosis of HL60 through activation of PARP cleavage. These effects, along with other impacts like inhibition of cell growth, cell migration, and induction of cell cycle arrests, suggest antileukemic potential of piperine when co-treated with curcumin	Li et al. (2020)
Breast cancer [Estrogen receptor negative cells (MDA-MB-231); estrogen positive cells (MCF-7)]	Piperidine derivative DTPEP synthesised from Tamoxifen (TAM)	Treatment with DTPEP in both MDA-MB-231 and MCF-7 marked cell cycle arrest in phase G_0_/G_1_ and ROS generation level were up-regulated in these cell lines. Mitochondrial cytochrome c was found to be released along with up-regulation of Bax and down-regulation of Bcl-2 and this high Bax:Bcl-2 ratio resulted in cell apoptosis. Treatment with DTPEP enhanced the expression level of ERß and down-regulated the expression of ERα (critical for the establishment of breast cancer). In MDA-MB-231 inhibition of the PI3K/AKT signaling pathway was observed through down-regulation of phosphorylation at Tyr485 of PI3k and Ser473 of AKT after treatment with DTPEP, all these effects show a great anti-breast cancer potential of piperidine	Arun et al. (2018)
Prostate cancer (PC3 cells)	Piperidine derivative Compound 17a	Treatment of PC3 cells with the piperidine derivative compound 17a results in an increase in the expression level of the Bax protein, while a decrease in the expression level of Bcl-2 and Bax, thus maintaining a high Bax:Bcl-2 ratio. These events result in PC3 cell apoptosis. Compound 17a treatment also affects the epithelial-mesenchymal transition and inhibits PC3 cell migration by upregulating E-cadherin and downregulating N-cadherin and Vimentin	Samykutty et al. (2013)
Colon cancer (DLD-1 and HT29 cells)	Piperidine derivative 2-amino-4-(1-piperidine) pyridine	Treatment of DLD-1 and HRT29 cells with this piperidine-derived compound inhibits cell cycle progression past phase S, thus arresting cell cycle in phase G_1_/G_0_. The specified treatment reduces the expression level of FOXA2 mRNA in DLD-1 and HT29 cells; this treatment also suppresses the epithelial mesenchymal transition (EMT) and down-regulates E-cadherin, and both these events inhibit the cell migration ability of DLD-1 and HT29 cells	Wang et al. (2019)
Lung cancer (H441 and A549 cells)	Piperidine derivative CLEFMA	CELFMA in lung cancer cells induces redox homeostasis, activates caspase 9/3 pathway through cleavage of PARP, which further leads to cell apoptosis. Furthermore, CELFMA also resulted in increased Bax release and decreased Bid release, thus maintaining a high Bax:Bid ratio, which is crucial for cancer cell apoptosis. P53 phosphorylation is also another result of CELFMA treatment-induced cell death, degradation of phosphorylated IκBα was also inhibited *via* proteasome inhibitors, thus restricting NF-κB from translocating into the nucleus. Cell inflammation was suppressed *via* decreasing the levels of pro-inflammatory cytokines along with COX-2 by CELFMA. ICAM1 responsible for cell adhesion, down-regulation of CD31 expression along with this G_1_/S phase transition of the cell cycle was also inhibited by CELFMA treatment	Yadav et al. (2013)
Ovarian cancer (OVCAR3 cells)	Piperidine derivative Tempol (TPL) and cisplatin (DDP)	Cotreatment of TPL and DPP resulted in few crucial anticancer effects on OVCCAR3 cells, such as inhibition of cell proliferation, increased apoptosis, and increased ROS generation. Further ROS accumulation by TPL and DPP leads to the release of mitochondrial cytochrome c and inducing a high Bax:Bcl-2 ratio which initiates the caspase-9/3 dependent apoptotic signalling pathway in OVCAR3 cells	Wang et al. (2018)

### Pharmacological Properties of Piperine and Piperidine

According to Vijayakumar et al. (2004) piperine offers possible protective activity against lipid peroxidation and antioxidant properties in rats fed a high-fat diet that causes oxidative stress in cells, (Vijayakumar et al., 2004). Piperine inhibits the P-glycoprotein, which increases the bioavailability of the flavonoid linarin in rats, and also aids cellular efflux during intestinal absorption (Feng et al., 2014). Be a result, piperine is referred to as a natural bio-enhancer (Dudhatra et al., 2012). Piperine causes a dose-dependent increase in stomach acid output and gastrointestinal motility disruption (Han, 2011). Piperine stimulates the liver, pancreas, and digestive enzymes in the small intestine mucosa when taken orally (Awen et al., 2010). Piperine may also boost protease, lipase, and pancreatic amylase activity when added to food components as a flavoring agents (Srinivasan, 2007). Piperine supplementation in conjunction with a high-fat diet lowered total cholesterol and body weight substantially thus it shows anti-obesity properties (Shah et al., 2011). In a dose-dependent way, piperine protected rats from PTZ-induced seizures. Piperine-treated rats had considerably different PTZ-induced convulsions than saline-treated animals, this implicates its anti-convulsion properties (Bukhari et al., 2013). In field of anti-asthmatic piperine contributes as it has been reported that compared to the control group of mice, the piperine-treated group exhibited reduced eosinophil infiltration, allergen - induced inflammation, and airway hyper-responsiveness through inhibition of the synthesis of interleukin-4, interleukin-5, immunoglobulin E, and histamine (Kim and Lee, 2009).

Piperidine alkaloids from *P. retrofractum* Vahl. (PRPAs) treatment was found to reduce adipocyte size along with this elevated serum levels of total cholesterol, low-density lipoprotein cholesterol, total lipid, leptin, and lipase were reduced by PRPA treatment. PRPA also reduced hepatic lipid buildup, which protected against the development of nonalcoholic fatty liver. Furthermore, PRPA increased AMPK signaling and changed the expression of proteins involved in lipid metabolism in the liver and skeletal muscle (Kim et al., 2011). *Picea abies* (L.) Karsten’s needles and bark contain the most piperidine alkaloid, epidihydropinidine which further was found to be inhibiting the growth of bacterial and fungal strains (Fyhrquist et al., 2017).

### Application of Nanotechnology

The pharmacological activities of piperine are limited due to its low water-solubility, fast metabolism, and systemic elimination (Pachauri et al., 2015; Gorgani et al., 2017). Besides, poor bioavailability of piperine are two factors that limit its efficacy as an anticancer agent. On the other hand, high concentrations of piperine were found to be toxic in animals (Chinta et al., 2017). However, these limitations (lower solubility and bioavailability vs. toxicity at higher doses) can be overcome by developing nanosystems such as liposomes, micelles, metal nanoparticles nanofibers, polymeric nanoparticles, and solid-lipid nanoparticles (Quijia and Chorilli, 2021). In order to enhance the solubility, piperine was incorporated in polymeric nanocapsules that demonstrated higher stability, controlled release and improved bioavailability (Rani et al., 2020). A recent study by (Thao et al. 2021) fabricated nanoliposomal complexes of piperine and anti-CD133 monoclonal antibodies (PMC). They proved very effective in inhibiting the growth of NTERA-2 cancer stem cells (CSCs) while being nontoxic to normal cells. In another study, aptamer conjugated piperine encapsulated polyethylene glycol-Polylactide-co-glycolide nanoparticles (P-PEG-PNP) and paclitaxel showed remarkable decline in needed paclitaxel dosages for suppressing the growth of paclitaxel resistant MCF-7 cells. Aptamer conjugated P-PEG-PNP could be applied as an efficacious and a safe nanoformulation for adjuvant cancer-chemotherapy targeting drug resistant breast cancers (Pachauri et al., 2015). This technology will greatly improve the efficacy and solubility of both piperidine and piperine as anticancer agents for future use.

## Discussion on Anti-Cancer Mechanisms of Piperine and Piperidine

**FIGURE 6:**
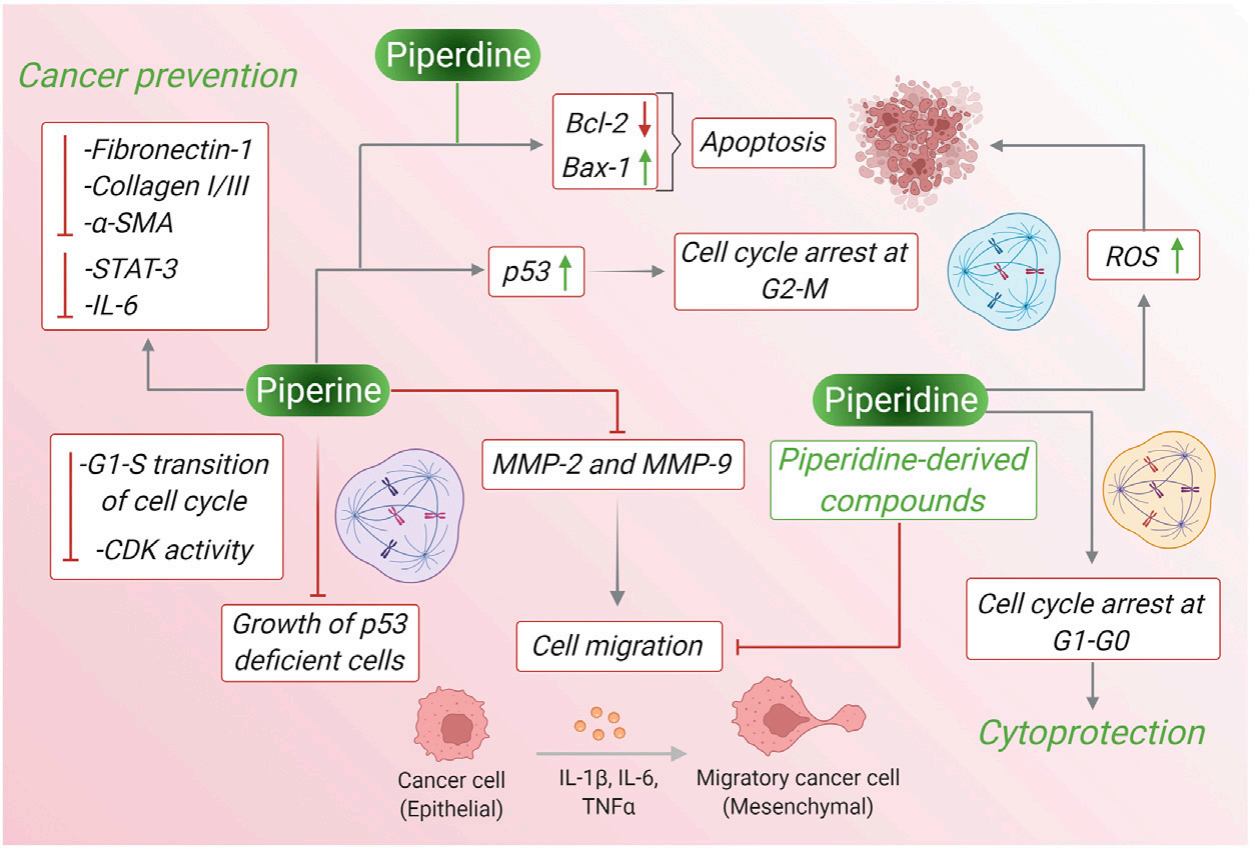
The pharmacological properties of piperine and piperidine against cancer comparing their molecular mechanisms and involved pathways.

Piperine and piperidine extracted from black pepper have been reported to have several anticancer properties. Often these two products act similarly to inhibit the survival, growth, and differentiation of cancer cell lines *via* several pathways like ROS generation, intrinsic and extrinsic caspase-mediated apoptotic pathway, inhibition of cancer cell migration, suppression of oncogene expression, increase synthesis of mitochondrial-mediated cytochrome c, BAX-2, and many more pathways. In case, of breast cancer piperine reduces phosphorylation of Ser473 residue in Akt signaling pathway whereas piperidine down-regulates phosphorylation at Tyr485 of PI3k and Ser473 of AKT signaling pathway leading to apoptosis. Piperine reduces the level of Bcl-2 and increases the level of Bax-2 and this high Bax:Bcl-2 ratio leading to initiation of caspase 9/3 dependent apoptosis in lung cancer cells and piperidine along with maintaining high Bax:Bcl-2 ratio, it also down-regulates CD31 expression and inhibits the G_1_/S phase transition of the cell cycle. In prostate cancer cells piperidine treatment inhibits PC3 cell migration by upregulating E-cadherin and downregulating N-cadherin and Vimentin, whereas piperine treatment to prostate cancer cells shows other activities like activating the caspase-3-dependent apoptotic pathway, suppressing phosphorylated STAT-3, down-regulation of the expression of Nf-κB, inducing PARP-1 cleavage and inhibition of B16-F10 invasion. Therefore, by different mechanism of actions piperine and piperidine leads to inhibition of several kinds of cancer. Figure 6 depicts the pharmacological properties of both the compounds against cancer comparing their molecular mechanisms and involved pathways.

## Conclusion

To date, natural products and phytochemicals have attracted great interest as an anticancer clinical and therapeutic agent among all pharmaceutical industries (Anand et al., 2019; Anand et al., 2021; Bandopadhyay et al., 2021; Das et al., 2021; Paul et al., 2021; Shoshan-Barmatz et al., 2021; Mitra et al., 2022). Although several preclinical reports have suggested the anticancer potential of black pepper-derived piperine and piperidine against several types of cancer, yet these phytochemicals have not attracted much attention from pharmaceutical industries; this may be due to poor aqueous solubility and less bioavailability of piperine and piperidine. Piperine supramolecular formulation with some hydrophilic compounds, such as unmodified cyclodextrin (CD) excipients, might ameliorate the condition (Ezawa et al., 2016; Ezawa et al., 2018). In China, piperine has been used clinically as an antiepileptic drug. As a result, if significant doses of piperine-containing herbal medications or foods are consumed in a single day for an extended length of time, unfavourable side effects may occur due to the varied pharmacological actions of piperine (Lee et al., 1984). The most palatable amount of piperine-containing black pepper revealed was 80 mg, when incorporated in a single serving of cereals to provide an enormous amount of food products to improve hypertension and lipid profile of an individual, and the outcomes between all sensory characteristics are extremely significant (Rashid et al., 2019). However, prior knowledge of these two chemicals, piperine and piperidine, and their contribution to a novel drug delivery system could be crucial before initiation of its clinical studies. After all of the study, piperine and piperidine could be considered as a novel anticancer drug, and it could be used as an alone therapeutic agent or in combination with preexisting conventional drugs in a dose-dependent manner. Finally, to introduce piperine and piperidine as an anticancer drug, more clinical studies are needed to improve and better describe its therapeutic action and efficiency as an anticancer agent.

## Glossary

AD: Androgen dependent
AI: Androgen independent
AKT: protein kinase B
AR: Androgen receptor
Bax: Bcl-2 associated X
Bcl-2: B-cell lymphoma 2
CDH1: Cadherin 1
CDK: cyclin-dependent kinase
CLEFMA: 4–[3,5-bis(2-chlorobezylidene)-4-oxo-piperidine-1-yl]-4-oxo-2-butenoic acid
COX2: Cyclooxygenase-2
CP: Chronic pancreatitis
c-SRC: SRC proto-oncogene, non-receptor tyrosine kinase
Cyto C: cytochrome C
DCFH-DA: 2′,7′-dichlorodihydrofluorescein diacetate
DDP: Cisplatin
DTPEP: piperidine derivative 1-(2-(4-(Dibenzo[b,f]thiepin-10-yl)phenoxy)ethyl)piperidine
EMT: Epithelial-mesenchymal transition
ER: Estrogen receptor
ERK1/2: Extracellular signal-regulated kinases
FDA: U.S. Food and Drug Administration
GBM: Glioblastoma multiforme
HRT18: Human rectal adenocarcinoma
IAP: Inhibitor of apoptosis
IL-1ß: Interleukin 1 beta
IL-6: Interleukin-6
IκBα: nuclear factor of kappa light polypeptide gene enhancer in B-cells inhibitor, alpha
JNK: c-Jun N terminal kinase
MACC1: Metastasis associated in colon cancer 1
MAD: Mother against decapentaplegic
MAPK: Mitogen-activated protein kinase
MDR: Multi drug resistant
MMC: Mitomycin-C
MMP-9/2: matrix metallopeptidase 9/2
MTT assay: 3-(4,5-dimethylthiazol-2-yl)-2,5-diphenyl-2H-tetrazolium bromide
NAC: N-Acetyl cysteine
NDRG2: NMYC downstream-regulated gene 2
NF-κB: nuclear factor kappalight-chain-enhancer of activated B cells
OSE: ovarian surface epithelial
PARP: poly ADP ribose polymerase
PGE2: Prostaglandin E2
PI3K: phosphatidylino sitol-3-kinase
PIP: Piperine
PSC: Pancreas Stellate cells
RhoA: Ras Homolog family member A
ROCK: Rho-Associated protein kinase
ROS: reactive oxygen species
SMAC/DIABLO: Second mitochondria-derived activator of caspase/direct inhibitor of apoptosis-binding protein with low pI
SMAD: Sma genes and the Drosophila
STAT3: signal transducer and activator of transcription 3
TAM: Tamoxifen
TGF-ß: Transforming growth factor beta
TMZ: Temozolomide
TNBC: Triple-Negative breast cancer
TPL: Tempol
VEGF: vascular endothelial growth factor
